# An HuR mutant, HuR-V225I, identified in adult T-cell Leukemia/Lymphoma, alters the pro-apoptotic function of HuR

**DOI:** 10.1038/s41420-024-02268-w

**Published:** 2024-12-18

**Authors:** Bianca Colalillo, Sujitha Sali, Ali H. Aldouhki, Isabelle Aubry, Sergio Di Marco, Michel L. Tremblay, Imed E. Gallouzi

**Affiliations:** 1 Department of Biochemistry, McGill University, Montreal, QC Canada; 2 Rosalind & Morris Goodman Cancer Institute, McGill University, Montreal, QC Canada; 3 KAUST Smart-Health Initiative and Biological and Environmental Science and Engineering (BESE) Division, King Abdullah University of Science and Technology (KAUST), Jeddah, Saudi Arabia

**Keywords:** RNA-binding proteins, Apoptosis

## Abstract

The RNA-binding protein HuR regulates various cellular processes, such as proliferation, differentiation, and cell fate. Moreover, recent studies have shown that HuR modulates the expression of factors important for tumor growth and progression. Despite its prominent role in tumorigenesis, until recently, there have been no reported mutations in HuR that have been associated to cancer. Here, we show that a HuR mutation, HuR-V225I, previously identified in a patient with Adult T-cell Leukemia/Lymphoma, interferes with the pro-apoptotic function of HuR. In response to apoptosis, HuR translocates to the cytoplasm and is cleaved in a caspase-dependent manner. In cervical cancer cells, neuroblastoma cells, and T-lymphocytes, we observed a decrease in cleavage of the HuR-V225I mutant under apoptotic conditions. This effect was shown to be mediated by the nuclear retention of HuR-V225I. Finally, expression of the HuR-V225I mutant decreases the cell’s response to apoptotic stimuli through the increased expression of mRNAs encoding anti-apoptotic factors, such as *XIAP* and *BCL-2*. Therefore, our data establishes that the absence of HuR cytoplasmic translocation and cleavage promotes cell viability, and that acquiring this mutation during tumorigenesis may thus reduce the efficacy of cancer therapy.

## Introduction

Post-transcriptional gene regulation is crucial for determining the fate of mRNA transcripts and is mediated by microRNAs, lncRNAs, and RNA-binding proteins (RBPs) [1–4]. HuR, a ubiquitously expressed RBP, interacts with AU-rich elements in the 3′ untranslated region (3′UTR) of its target transcripts, influencing their stability and translation [4–8]. Importantly, HuR has the ability to shuttle between the nucleus and cytoplasm in response to certain stimuli [6, 7, 9]. HuR modulates cellular processes, including differentiation [10, 11], cell proliferation [12, 13], and cell fate [14–16], and is implicated in disease progression, particularly cancer [4]. In many cancers, HuR is broadly upregulated and abundant in the cytoplasm [6, 17–20], whereby it carries out its post-transcriptional function and regulates transcripts involved in tumorigenic processes such as cell cycle regulation, angiogenesis, and metastasis [9, 21, 22].

Although the role of HuR in cancer has been established, until recently, there have been no reported Single Nucleotide Polymorphisms (SNPs) in the HuR gene (*ELAVL1*) associated with cancer onset in patients. In 2015, Kataoka et al. identified an extensive list of cellular genes and pathways commonly altered in Adult T-cell Leukemia/Lymphoma (ATLL), as well as novel mutations not previously defined [23]. While their findings identified genetic alterations in T-cell signaling pathways, many of the newly described mutational targets in their supplemental data have not been thoroughly investigated. Interestingly, one of the mutations reported is a mutation in HuR, HuR-V225I, which was identified in one patient with ATLL. This mutation is of significance because it is found within the HuR nucleocytoplasmic shuttling sequence (HNS) in the hinge region of HuR. In addition to playing a role in regulating the subcellular localization of HuR, the HNS has also been shown to be crucial for the pro-apoptotic function of HuR [14, 24].

We previously showed that in response to lethal stress, HuR is exported to the cytoplasm in association with the apoptosome activator, PP32/PHAP1. HuR is subsequently cleaved by caspase-3 and -7 at Aspartate (D) 226, generating two cleavage products (HuR-CP1 and HuR-CP2) [14]. This cleavage event promotes the activation of caspase-mediated apoptosis in which: CP2 interacts with PP32/PHAP1 to activate the apoptosome, HuR accumulates in the cytoplasm due to CP1 sequestering its import factor, Transportin-2 (TRN2), and both CP1 and CP2 bind to and stabilize the pro-apoptotic *caspase-9* transcript [11, 15, 25]. Given that the newly identified HuR mutation, HuR-V225I, is found in the residue before HuR’s cleavage residue (D226A), we sought to investigate the impact of the HuR mutation, V225I, on the pro-apoptotic function of HuR. We established that the V225I mutant is cleaved less and remains nuclear in response to apoptotic stress. Moreover, the disruption of HuR cleavage by the presence of the V225I mutation decreases the cell’s response to apoptosis through the increased expression of anti-apoptotic factors.

## Results

### HuR-V225I prevents apoptosis-induced HuR cleavage in cancer cells

Previously published work has highlighted the importance of HuR cleavage at D226 for its pro-apoptotic function [14]. Hence, we first sought to assess the effect of the HuR-V225I mutation on the cleavage of HuR. HeLa cervical cancer cells were treated with drugs, such as staurosporine (STS), that were previously shown to induce apoptosis [14, 15, 25, 26]. STS-treated HeLa cells exhibit classical morphological signs of apoptotic cell death, as shown by their rounded and shrunken shape (Fig. S1A), and the striking increase in the percentage of cells undergoing nuclear blebbing (Fig. S1B). At the molecular level, treatment of HeLa cells with STS, but not the DMSO solvent control, induces the activation of apoptotic pathways, as evidenced by the cleavage of the pro-apoptotic marker PARP1 and caspase-3, an effector caspase (Fig. S1C, D). Notably, the cleavage product of endogenous HuR (CP1) is apparent within 1.5 h of STS treatment (Fig. S1C, D).

**Fig. 1:**
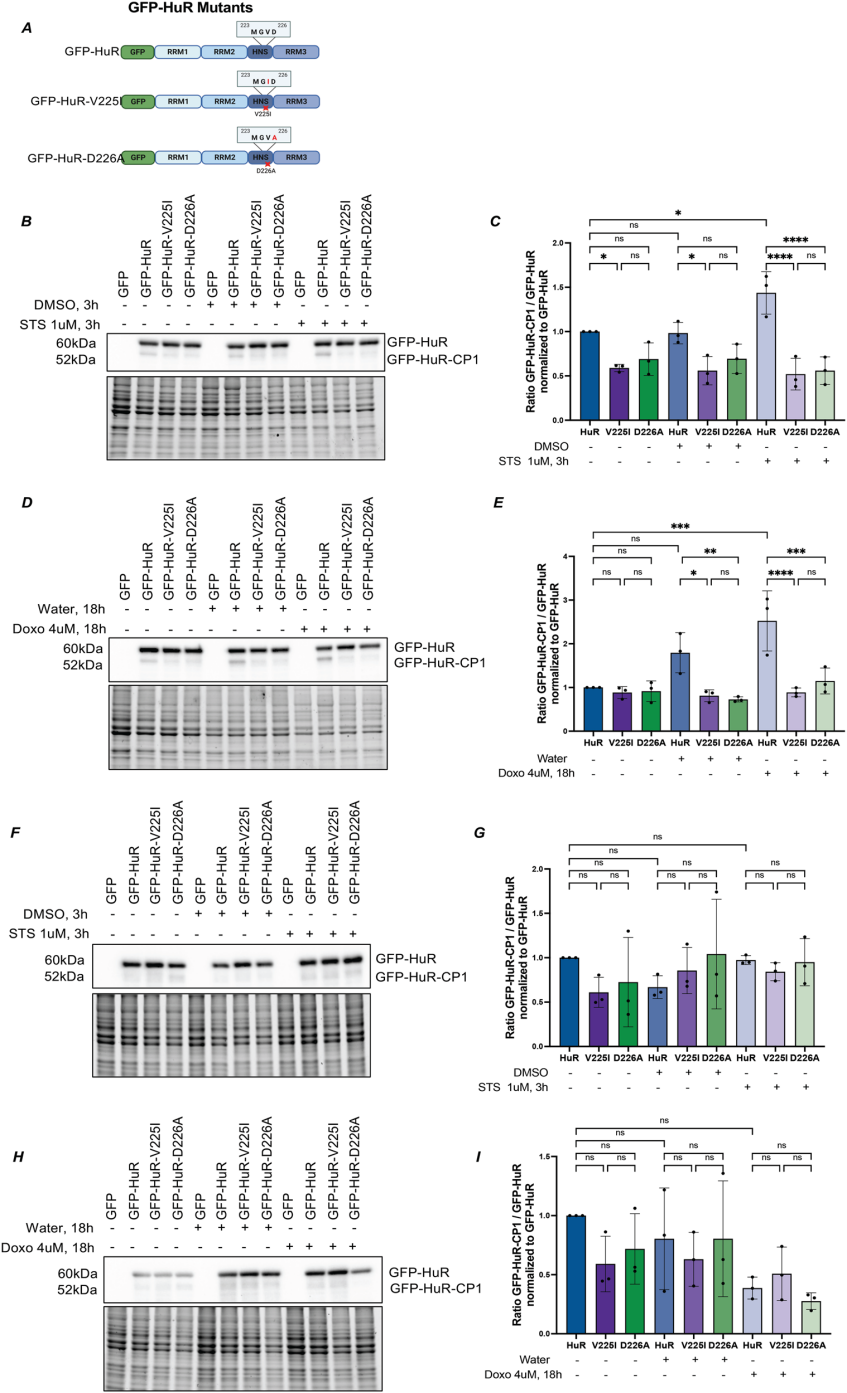
HuR-V225I is cleaved less than wild-type HuR in response to apoptosis. **A** GFP-HuR fusion proteins. N-terminal GFP fused to either wild-type HuR (top) or point mutants HuR-V225I and HuR-D226A (middle and bottom, respectively). **B** Western blot analysis of HeLa cells transfected with GFP or GFP-HuR fusion proteins treated with 1 μM STS or equivalent volume of DMSO for 3 h. Immunoblotting was performed using anti-HuR. Total protein content (bottom) was used as loading control. **C** Quantification of the ratio of cleavage products of GFP-HuR fusion proteins in (**B**) to their full-length counterparts, normalized to the ratio obtained in untreated cells. **D** Western blot analysis of HeLa cells transfected with GFP or GFP-HuR fusion proteins treated with 4 μM Doxorubicin or equivalent volume of water for 18 h. Immunoblotting was performed using anti-HuR. Total protein content (bottom) was used as loading control. **E** Quantification of the ratio of cleavage products of GFP-HuR fusion proteins in (**D**) to their full-length counterparts, normalized to the ratio obtained in untreated cells. **F** Western blot analysis of HEK-293T/17 cells transfected with GFP or GFP-HuR fusion proteins treated with 1 μM STS or equivalent volume of DMSO for 3 h. Immunoblotting was performed using anti-HuR. Total protein content (bottom) was used as loading control. **G** Quantification of the ratio of cleavage products of GFP-HuR fusion proteins in (**F**) to their full-length counterparts, normalized to the ratio obtained in untreated cells. **H** Western blot analysis of HEK-293T/17 cells transfected with GFP or GFP-HuR fusion proteins treated with 4 μM Doxorubicin or equivalent volume of water for 18 h. Immunoblotting was performed using anti-HuR. Total protein content (bottom) was used as loading control. **I** Quantification of the ratio of cleavage products of GFP-HuR fusion proteins in (**H**) to their full-length counterparts, normalized to the ratio obtained in untreated cells. Error bars represent means and ±SEM of at least three independent experimental replicates with *P ≤ 0.05, **P ≤ 0.01, ***P ≤ 0.001, and ****P ≤ 0.0001. ns not significant by one-way ANOVA.

To evaluate the effect of the HuR-V225I mutation on the pro-apoptotic cleavage of HuR, we transfected HeLa cells with N-terminal GFP-HuR fusion proteins (Fig. 1A): GFP fused to wild-type HuR (GFP-HuR), GFP fused to the HuR-V225I point mutant (GFP-HuR-V225I) and GFP fused to the previously published non-cleavable HuR point mutant, HuR-D226A (GFP-HuR-D226A) [14]. We confirmed, as expected, that GFP-HuR, but not the GFP-HuR-D226A mutant, is cleaved in STS-treated HeLa cells (Fig. 1B, C). Similarly, the GFP-HuR-V225I mutant is also not cleaved in these cells (Fig. 1B, C). We noticed a slight accumulation of cleavage product of wild-type GFP-HuR, but not the mutants, in the absence of STS, which is most likely due to background apoptosis brought on by transfection of the GFP-HuR plasmid (Fig. 1B, C).

Interestingly, the effect of the V225I mutation on the cleavage of HuR is not specific to the treatment of cells with STS, nor to cervical cancer cells undergoing apoptosis. Indeed, while the cleavage of GFP-HuR was observed in HeLa cells treated with the apoptosis-inducing chemotherapeutic drug doxorubicin (Figs. S1D and 1D, E) or in neuroblastoma cells treated with STS (Fig. S2), these effects are negated due to the V225I mutation.

Finally, given that the HuR-V225I mutation was identified in cancer cells, we sought to assess whether this cleavage effect was cancer cell specific. The cleavage of HuR and the subsequent effect of the V225I mutation is not observed in normal HEK-293T/17 cells treated with STS or Doxo (Fig. 1F–I). Together, these observations suggest that similar to the D226A mutation, the V225I mutation also prevents the caspase-mediated cleavage of HuR [14, 15] under apoptotic conditions. Additionally, this effect is shown to be cancer relevant but not cell line specific, given that these results were observed in multiple cancer cell lines but not in normal cells.

### HuR-V225I decreases the cytoplasmic translocation of HuR in response to apoptosis

**Fig. 2:**
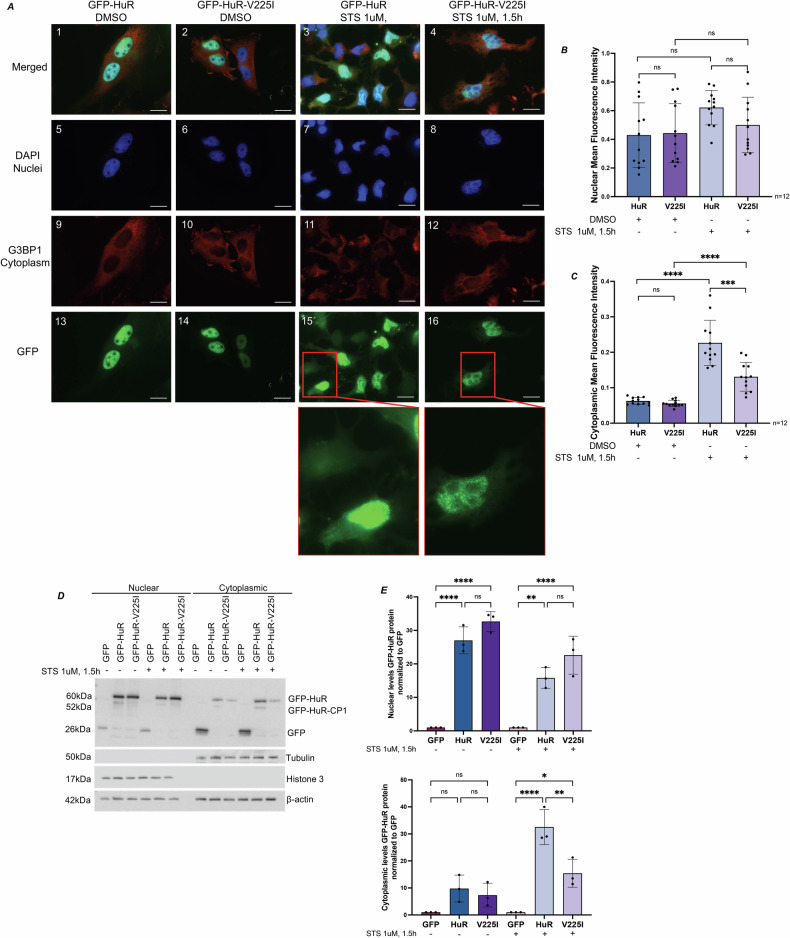
HuR-V225I remains predominantly nuclear in response to STS-induced apoptosis in HeLa cells. **A** Representative immunofluorescent images of HeLa cells transfected with GFP-HuR or GFP-HuR-V225I, treated with 1 μM STS or equivalent volume of DMSO for 1.5 h. Cells were stained with DAPI (nuclei), G3BP1 (cytoplasm) and GFP (HuR fusion proteins). Magnification 63×. **B** Quantification of nuclear mean fluorescence intensities of GFP-HuR vs. GFP-HuR-V225I in DMSO and STS-treated conditions. **C** Quantification of cytoplasmic mean fluorescence intensities of GFP-HuR vs. GFP-HuR-V225I in DMSO and STS-treated conditions. **D** Western blot analysis of subcellular fractionation assay of HeLa cells transfected with GFP-HuR or GFP-HuR-V225I, with and without 1 μM STS treatment for 1.5 h. Immunoblot for GFP, Histone 3 (nuclear marker) and Tubulin (cytoplasmic marker). **E** Quantification of GFP-HuR fusion proteins in nuclear vs. cytoplasmic fractions. Protein levels are normalized to β-actin and quantification is relative to GFP-transfected cells. Nuclear and cytoplasmic mean fluorescence intensities of GFP-HuR fusion proteins were quantified using Human C–N Translocation pipeline from Cell Profiler Software. Error bars represent means and ±SEM of at least three independent experimental replicates with *P ≤ 0.05, **P ≤ 0.01, ***P ≤ 0.001, and ****P ≤ 0.0001. ns, not significant by one-way ANOVA.

The hinge domain of HuR is essential for regulating its subcellular localization, as it contains the HNS [24, 27]. It has been well-established that the cytoplasmic accumulation of HuR is required for its cleavage [10, 14, 15, 25]. We thus assessed whether the decrease in GFP-HuR-V225I cleavage is due to changes in its subcellular localization. Using immunofluorescent microscopy, we observed no changes in the localization of the GFP-HuR-V225I mutant compared to wild-type GFP-HuR under non-treated (Fig. S3) and solvent-treated conditions (Fig. 2A panels 13,14, B, C). As expected, GFP-HuR showed significant cytoplasmic accumulation in response to 1.5 h of STS treatment (Fig. 2A panels 3, 15, B, C). In contrast, GFP-HuR-V225I remains predominantly nuclear under apoptotic conditions (Fig. 2A panels 4, 16, B, C). Similar to the GFP-HuR-V225I mutant, the D226A mutant remains nuclear in response to STS-induced apoptosis (Fig. S4). To further validate these results, we performed a subcellular fractionation assay to determine, by western blot, the localization of the GFP-HuR-V225I mutant in response to STS. We observed that while there is a trend in the increased accumulation of the V225I mutant in the nucleus relative to wild-type HuR, there is a significant decrease in the accumulation of the HuR-V225I mutant in the cytoplasm in STS-treated conditions compared to wild-type GFP-HuR (Fig. 2D, E). Overall, this data suggests that the nuclear retention caused by the V225I mutation prevents the generation of pro-apoptotic cleavage products.

### Expression of HuR-V225I in HeLa cells decreases their response to apoptosis

**Fig. 3:**
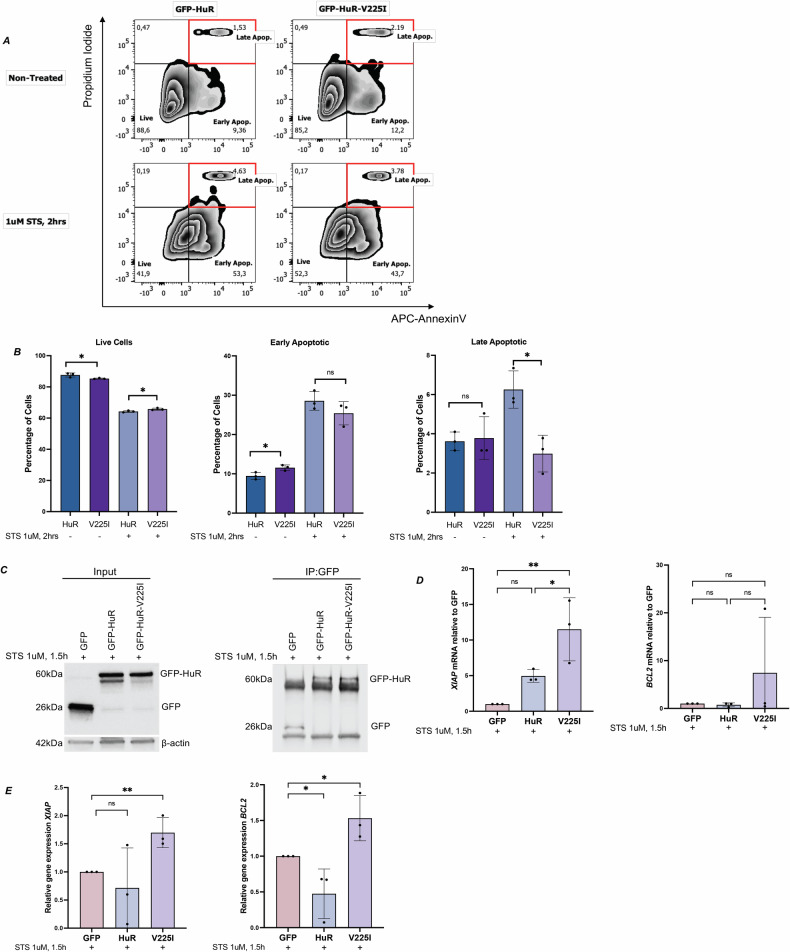
GFP-HuR-V225I exhibits anti-apoptotic effect in HeLa cells. **A** Representative figures of flow cytometric analysis of HeLa cells transfected with GFP-HuR or GFP-HuR-V225I, with and without 1 μM STS treatment for 2 h. Cells were stained with APC-AnnexinV and Propidium Iodide to assess apoptosis. **B** Percentages of live cells (left), early apoptotic cells (middle), and late apoptotic cells (right). **C** (Left) Western blot analysis following immunoprecipitation of GFP, GFP-HuR and GFP-HuR-V225I in response to 1 μM STS for 1.5 h. Total levels of these proteins were assessed in Input (Right). **D** *XIAP* and *BCL-2* mRNA levels associated with immunoprecipitated GFP-HuR or GFP-HuR-V225I. Relative gene expression was normalized to GFP control. **E** *XIAP* and *BCL-2* mRNA levels in HeLa cells transfected with GFP, GFP-HuR or GFP-HuR-V225I treated with 1 μM STS for 1.5 h. Gene expression levels are normalized to GFP-transfected cells. Error bars represent means ± SEM of at least three independent experimental replicates with *P ≤ 0.05, **P ≤ 0.01, ns, not significant by Unpaired t-test.

Next, we sought to assess the functionality of the V225I mutation on the pro-apoptotic function of HuR. We used flow cytometric analyses to determine the percentage of cells undergoing apoptosis upon the expression of wild-type HuR or its V225I counterpart. HeLa cells transfected with either GFP-HuR or GFP-HuR-V225I and treated with 1 μM STS for 2 h were stained with AnnexinV and Propidium Iodide (PI) and analyzed by flow cytometry (Fig. 3A). Live cells are both APC-AnnexinV and PI negative and apoptotic cells are double positive. The percentage of GFP-HuR-V225I expressing live cells is slightly higher than those expressing GFP-HuR in response to STS treatment (Fig. 3B, left). The percentages of STS-treated cells undergoing late stages of apoptosis are considerably decreased when expressing the V225I mutant (Fig. 3B, right).

Moreover, to uncover the mechanism through which HuR-V225I contributes to increased cell survival, we assessed HuR-V225I association with previously identified anti-apoptotic HuR targets, *XIAP* and *BCL-2*. RNA immunoprecipitation and RT-qPCR analyses revealed that the association of HuR-V225I with the *XIAP* mRNA is significantly greater than that of wild-type HuR. Although not significant, HuR-V225I also showed a trend toward increased association with the *BCL-2* mRNA (Fig. 3D). Additionally, in response to STS, the overexpression of the V225I mutant increases the expression of the *XIAP* and *BCL-2* mRNAs (Fig. 3E). Collectively, these results indicate that HuR-V225I associates with and increases the expression of anti-apoptotic factors, *XIAP* and *BCL-2*, under apoptotic conditions, thus promoting cell survival.

### HuR-V225I prevents apoptosis-induced HuR cleavage in T-lymphocytes

**Fig. 4:**
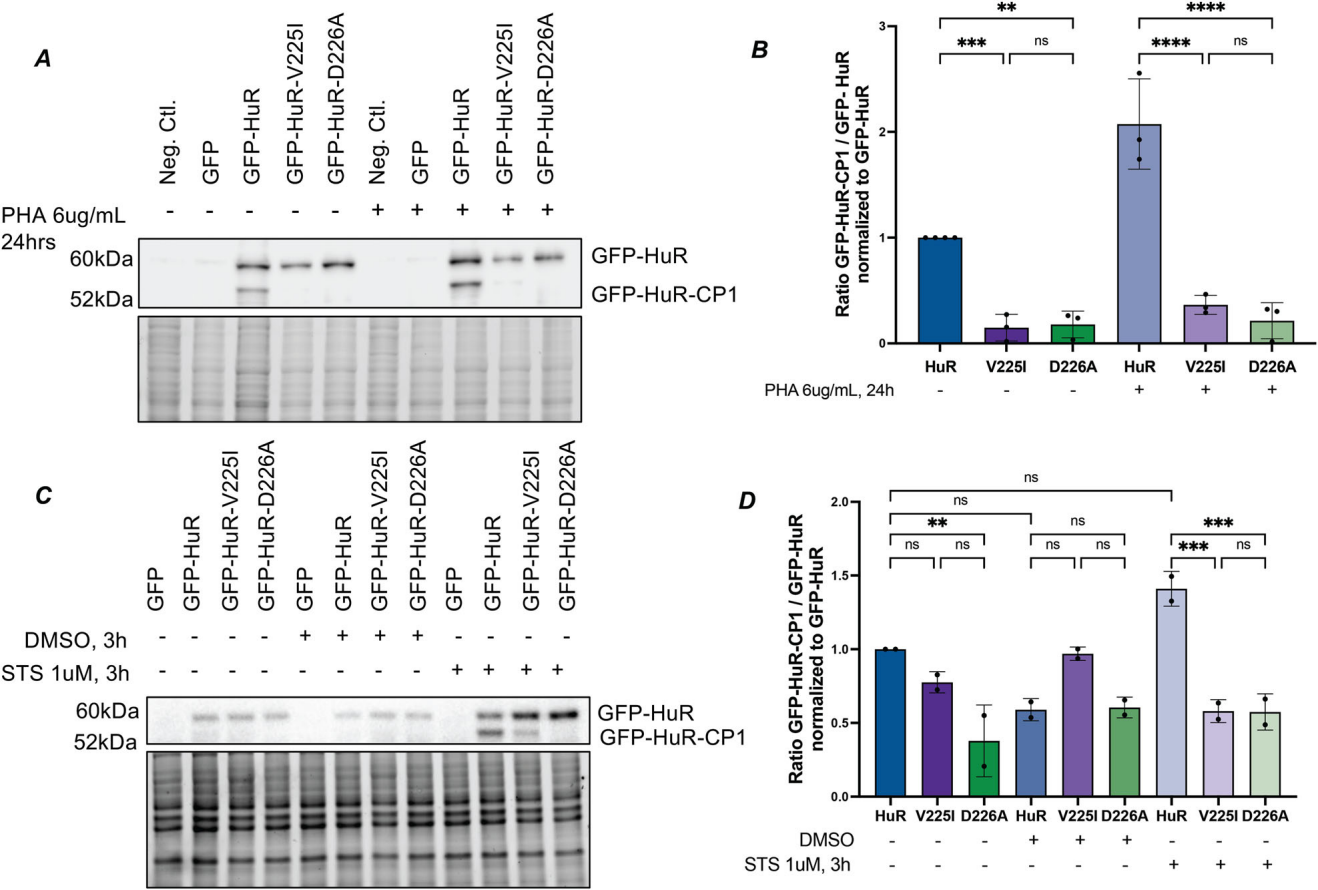
HuR-V225I is cleaved less than wild-type HuR in response to apoptosis in T-lymphocytes. **A** Western blot analysis of Jurkat T-lymphocytes transfected with GFP or GFP-HuR fusion proteins with and without 6 μg/mL PHA treatment for 24 h. Immunoblotting was performed using anti-HuR. Total protein content (bottom) was used as loading control. **B** Quantification of the ratio of cleavage products of GFP-HuR fusion proteins in (**A**) to their full-length counterparts, normalized to the ratio obtained in untreated cells. **C** Western blot analysis of Molt-4 T-lymphocytes transfected with GFP or GFP-HuR fusion proteins with 1 μM STS or equivalent volume of DMSO for 3 h. Immunoblotting was performed using anti-HuR. Total protein content (bottom) was used as loading control. **D** Quantification of the ratio of cleavage products of GFP-HuR fusion proteins in (**C**) to their full-length counterparts, normalized to the ratio obtained in untreated cells. Error bars represent means and ± SEM of at least three independent experimental replicates with *P ≤ 0.05, **P ≤ 0.01, ***P ≤ 0.001, and ****P ≤ 0.0001. ns not significant by one-way ANOVA.

To uncover the role of this mutation in a system that is physiologically relevant to Adult T-cell Leukemia/Lymphoma, we established a model of apoptotic cell death in T-lymphocytes. Apoptosis was induced in Jurkat and Molt-4 T-lymphocytes using phytohemagglutinin (PHA) and STS, respectively. PHA is a lectin that, when bound to the surface of T-cells, induces cell death through the expression of the pro-apoptotic Fas death receptor [28, 29]. Jurkat cells were treated with 2–10ug/mL of PHA for 24 h to confirm that PHA induces apoptosis. At the molecular level, PHA treatment promotes the activation of apoptotic pathways, as shown by the increase in PARP1 and caspase-3 cleavage. There is also a dose-dependent increase in the cleavage of endogenous HuR in response to PHA (Fig. S5A, B). Similarly, treatment of Molt-4 T-lymphocytes with STS induces apoptosis, as evidenced by the cleavage of PARP1, caspase-3, and HuR (Fig. S5C, D), whereas solvent-treated controls do not exhibit an effect. To demonstrate the lack of GFP-HuR-V225I cleavage in T-lymphocytes, we transfected Jurkat or Molt-4 cells with N-terminal GFP-tagged HuR fusion proteins (Fig. 1A) and treated them with 6ug/mL PHA for 24 h or 1 μM STS for 3 h. As expected, wild-type GFP-HuR, but not the V225I mutant, is cleaved in response to apoptosis in both Jurkat and Molt-4 T-lymphocytes (Fig. 4A–D). Similar to the HeLa cells, we observed a minor accumulation of cleavage product of wild-type GFP-HuR in non-treated conditions, due to the transfection of the GFP-HuR plasmid in Jurkat cells (Fig. 4A). In summary, we demonstrate that the HuR-V225I mutant is more resistant to pro-apoptotic caspase-mediated cleavage in T-lymphocytes.

## Discussion

**Fig. 5:**
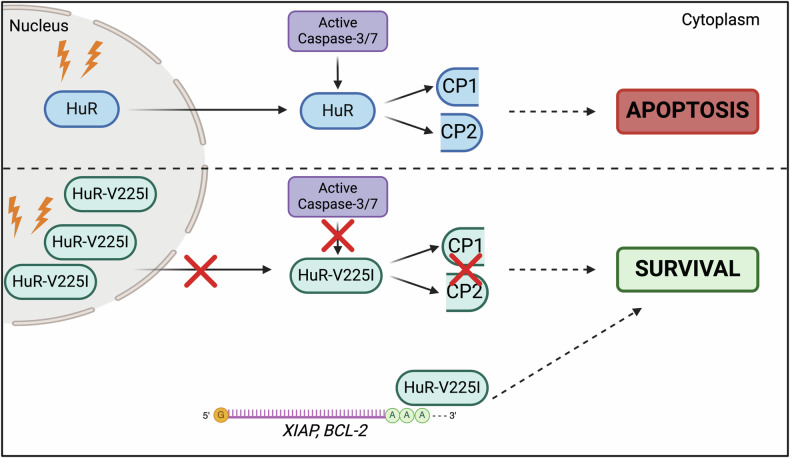
Schematic model depicting HuR-V225I pro-survival function. In response to apoptotic stimuli, wild-type HuR has been shown to translocate to the cytoplasm and undergo cleavage at D226 mediated by caspase-3 and -7. This cleavage event contributes to driving cell death. However, the HuR-V225I mutant, in response to apoptotic stimuli, remains nuclear and is thus not cleaved. The disruption of cleavage results in increased cellular survival through its increased association with anti-apoptotic mRNAs, *XIAP* and *BCL-2*. Figure created with BioRender.com.

In this study, we investigated the impact of a HuR mutation, previously identified in a patient with Adult T-cell Leukemia/Lymphoma, on the pro-apoptotic function of HuR. Our data demonstrates that the HuR-V225I mutation, located in the caspase-cleavage site of HuR, interferes with its pro-apoptotic function. In contrast to wild-type GFP-HuR, the V225I mutation in HuR prevents its pro-apoptotic cleavage in cancer cells. Moreover, this effect was shown to be cancer relevant, as the cleavage of HuR or HuR-V225I was not observed in HEK-293T/17 embryonic kidney cells. We have previously shown that the cytoplasmic accumulation of HuR is required for the production of its pro-apoptotic cleavage products [14, 15]. Using fluorescence microscopy and subcellular fractionation experiments, we demonstrated that the V225I mutant remained nuclear in response to apoptosis, thus preventing its caspase-mediated cleavage. Furthermore, we observed that the V225I mutant significantly decreased the percentage of STS-treated HeLa cells undergoing late stages of apoptosis. This pro-survival effect was found to be driven by the increased association of HuR-V225I with anti-apoptotic HuR targets, *XIAP* and *BCL-2* mRNAs, resulting in their increased levels. Our data corroborates the hypothesis that the mutation of the V225 residue in the cleavage site of HuR inhibits the production of cleavage products and promotes the expression of anti-apoptotic transcripts (Fig. 5). Thus, these findings demonstrate that mutations in the caspase-cleavage site of HuR in cancer cells may promote resistance to apoptotic cell death.

The significance of a pro-survival HuR mutant is especially critical in the context of cancer. The role of HuR in cancer has been extensively investigated and it has been identified as an important mediator of cancer progression through its upregulation, cytoplasmic accumulation and stabilization of pro-tumorigenic mRNAs [9, 21, 22]. However, the HuR-V225I mutation is the first SNP in HuR that has been associated with cancer in patients [23]. Our study has established that this mutation prevents the apoptosis-induced cytoplasmic translocation and cleavage of HuR in cancer cell lines. Moreover, this mutation increases cell survival by stabilizing the pro-apoptotic transcripts, *XIAP* and *BCL-2*. This is concurrent with existing evidence showing that preventing the cleavage or cytoplasmic translocation of HuR in cancer cells promotes cell viability [30–32]. Therefore, our data suggests that the V225I mutation is a poor prognostic factor in cancer. While the mutation itself does not confer full resistance to apoptosis, transformed cells that acquire this mutation are more likely to resist traditional cancer therapy through increased cell survival.

HuR has been shown to have both pro- and anti-apoptotic target transcripts in response to various stimuli. For instance, following its cleavage in response to lethal stress, HuR cleavage products, as well as full-length HuR, bind to and stabilize the pro-apoptotic *caspase-9* mRNA [14, 15, 33]. However, under homeostatic conditions or in response to other stimuli such as short-wavelength ultraviolet light or cellular polyamines, HuR has been shown to stabilize mRNAs encoding anti-apoptotic factors; *Survivin*, *BCL-2*, *MCL-1*, *Prothymosin-α* and *XIAP* [34–37]. Our data indicates that expressing the HuR-V225I mutant in HeLa cells in response to apoptotic stress promotes cell survival through the increased association and levels of anti-apoptotic mRNAs *XIAP* and *BCL-2*. XIAP, or X-linked inhibitor of apoptosis protein, is a well-known pro-survival factor that blocks the activation of apoptotic caspases, caspase-3, -7 and -9 [38]. Given that caspase-3 and caspase-7 cleave HuR [14], it is possible that the increased XIAP expression mediated by the HuR-V225I mutant is inhibiting these caspases and preventing the cleavage products from being formed. Moreover, recent studies have demonstrated that XIAP expression is upregulated in many cancers [38–41]. Importantly, XIAP overexpression has been correlated to increased malignant transformation, poor prognosis, and decreased response to cancer therapies [38–42]. Therefore, in accordance with our data, cancer cells expressing the V225I mutant, or another mutation in the caspase-cleavage site of HuR, would have increased XIAP expression and thus have an increased likelihood of resistance to cancer therapy.

Numerous studies have aimed to develop small molecular inhibitors that target HuR in cancer [9, 21, 22, 43, 44]. The majority of these compounds either block the interaction between HuR and its target transcripts or prevent its cytoplasmic accumulation [45–47]. Our findings, however, suggest that the lack of cytoplasmic accumulation may provide cancer cells with a pro-survival benefit. This opens the possibility of designing novel cancer therapies promoting the cytoplasmic accumulation of HuR in the context of chemotherapeutic treatments that induce apoptosis.

Our study has thus furthered our understanding of the role of HuR in apoptosis, specifically highlighting the importance of its caspase-mediated cleavage in driving cell death. Moreover, our results suggest, for the first time, that the presence of a mutation within the HuR cleavage site can predispose patients to have decreased response to cancer therapies.

## Materials and methods

### Reagents and antibodies

The following antibodies were used: PARP1 (SC-8007, SC-7150, CST#9542), Cleaved Caspase-3 (CST#9661, CST#9662, CST#9446), G3BP1 (CST#17798), Histone-3 (Abcam#AB8898), Tubulin (Abcam#AB11304), HuR 3A2 [48], GFP (Conetech#JL-8) and β-actin (Invitrogen#BA3R). The following reagents were used for immunoblotting: PVDF membranes (Immobilin-P#IPVH00005), HRP-conjugated goat secondary antibodies (Jackson ImmunoResearch#115-035-062 and #111-035-003), ECL (Perkin-Elmer#NEL122001EA). For FACS analysis: APC-Annexin-V (BioLegend#640920), AnnexinV Binding Buffer (BioLegend#422201) and Propidium Iodide (BioLegend#421301). The following reagents were used for immunofluorescence: DAPI (Fisher-Scientific#D1306), Mowiol-4-88 (Sigma-Aldrich#81381), AlexaFluor-594 conjugated goat-anti-rabbit antibody (Life-Technologies#A11037).

### Cell culture and transfection

HeLa (CRM-CCL-2), SH-SY5Y (CRL-2266), HEK-293T/17 (CRL-11268), Jurkat (Clone E6.1) and Molt-4 (CRL-1582) cells were purchased from American Type Culture Collection. All cell lines were grown on TC-treated dishes (Corning#C3516,#430167, #C430639,#430614U) in a 5% CO_2_ environment at 37 °C. Hela and SH-SY5Y were grown in DMEM/F12 (HyClone#SH3002301 and Gibco#11320-074) supplemented with 10% FBS (Life Technologies#12483020 and Gibco#10082-147) and 1% penicillin/streptomycin (HyClone#SV30010 and Gibco #15140-122). HEK-293T/17 were grown DMEM/F12 (HyClone#SH3002301) supplemented with 10% FBS (Life Technologies#12483020) and 1% Gentamicin (Wisent#450-135). Cells were transfected at 80% confluency and transfections were done as described by the Polyplus jetPRIME transfection protocol, using 0.5ug/mL plasmid. Jurkat and Molt-4 T-lymphocytes were grown in RPMI-1640 (HyClone#SH3002701) supplemented with 10% FBS (Life Technologies#12483020) and 1% penicillin/streptomycin (HyClone#SV30010). 1.25 × 10^6^ cells were transfected with 3 μg plasmid as described by the Lonza Bioscience Cell Line Nucleofector^TM^ Kit V (VCA-1003), using the X-005 program of the Nucleofector^TM^IIb System. Cells were routinely monitored for mycoplasma.

### Induction of apoptosis

Apoptosis was induced using Staurosporine (Sigma-Aldrich#S4400) (1 μM for 0.5–3 h), Doxorubicin (SelleckChem#E2516) (0–8 μM,18 h), or Phytohemagglutinin-M (PHA-M) (Millipore-Sigma#11082132001) (2–10 μg/mL, 24 h).

### Plasmid construction

N-terminal GFP-tagged plasmids were generated as previously described [14]. Wild-type GFP-HuR and point mutant (GFP-HuR-V225I and GFP-HuR-D226A) plasmids were constructed by NorClone Biotech Laboratories. Plasmids were purified using Geneaid Presto^TM^ Mini Plasmid Kit (#PDH300).

### Immunoblotting

Cells were washed twice with cold Phosphate-Buffered Saline (PBS) and lysed with RIPA buffer (150 mM NaCl, 50 mM Tris-HCl pH7.4, 1%NP-40, 0.5% Sodium Deoxycholate, 0.1% SDS) supplemented with 1× Protease-Inhibitor (Roche#04693159001) and 0.1 M Sodium-Orthovanadate on ice for 20 min. Protein lysates were collected following high-speed centrifugation and total protein content was quantified (Thermo Fisher Scientific#PI23225) and normalized amongst samples. Lysates were then diluted with Laemmli buffer. Proteins were resolved on acrylamide gels supplemented with 0.5% (v/v) tri-chloro-ethanol to visualize total protein loading. Following separation by SDS-PAGE, proteins were transferred to PVDF membranes using the Bio-Rad Trans-Blot Turbo^TM^ system. Membranes were blocked with a 2% polyvinyl alcohol (PVA) solution containing 0.05% Thimerosal for 2 min, then washed with PBS containing 0.1% Tween-20 (PBS-T). Membranes were incubated in primary antibodies diluted in 5% BSA/PBS-T as follows: PARP1(1:500), Cleaved-Caspase-3(1:1000), HuR(1:1000), Histone-3 (1:2000), Tubulin(1:1000), GFP(1:1000). HRP-conjugated secondary antibodies were diluted in 5% skim milk/PBS-T. Chemiluminescent signal was detected using the ChemiDoc^TM^ imaging system and analyzed digitally (ImageLab, Bio-Rad). Statistical analysis was performed using GraphPad Prism 10 software.

### Immunofluorescence microscopy

Immunofluorescence (IF) was performed 24 h after transfection of HeLa cells and 1.5 h after treatment with STS. HeLa cells were cultured in 6-well plates (Corning#C3516) lined with 18 mm glass coverslips (Fisherbrand#12-541A). Cells were washed twice with cold PBS and fixed with 4% Paraformaldehyde/PBS for 20 min at room temperature. Cells were then permeabilized (0.5%Triton X-100, 1% goat-serum, 1× PBS) for 15 min and blocked for 60 min at room temperature with Goat Serum Blocking Solution (2% goat-serum, 1%BSA, 0.1%Cold Fish Skin Gelatin, 0.1%Triton X-100, 0.05%Tween-20, 0.05%Sodium Azide, PBS). Cells were then incubated with primary antibody against G3BP1 (1:1000) overnight, washed, and incubated with AlexaFluor-594 conjugated goat-anti-rabbit secondary antibody (1:800) for 1 h. Cells were stained with DAPI for the last 10 min of secondary antibody incubation. Coverslips were then mounted onto microscope slides (Fisherbrand Superfrost Plus) with Mowiol4-88 mounting medium. Images were captured with Zeiss Observer.Z1 microscope and Zeiss AxioCam-MRm digital camera. Nuclear and cytoplasmic mean fluorescent intensities were quantified using the Human C-N Translocation Assay from CellProfiler software [49, 50]. Statistical analysis was performed using GraphPad Prism V.10 software.

### Subcellular fractionation

Subcellular fractionation experiments were performed as previously described [51]. Cells were washed twice with cold PBS, lysed in EBKL buffer (25 mM HEPES, pH7.6, 5 mM MgCl2, 5 mM KCl, and 0.5%NP-40) on ice for 15 min, then further lysed by 35 strokes with the tight pestle of a Dounce-type homogenizer. Following a series of low-to-high-speed centrifugations, the cytoplasmic fraction was separated from the nuclear pellet. The nuclear pellet was then washed three times with EBMK buffer (25 mM HEPES, pH7.6, 5 mM MgCl2, 1.5 mM KCl, 75 mM NaCl and 175 mM sucrose) and resuspended in water containing 0.5%NP-40. Lysates were diluted with Laemmli buffer and analyzed by western blot, as described above.

### APC-AnnexinV/propidium iodide assay

APC-AnnexinV/propidium iodide assay of plasmid transfected HeLa cells was performed 24 h after transfection and 2 h after treatment with STS. Cells were detached with Trypsin, centrifuged (300 × *g*, 5 min) and resuspended in PBS. Cells were counted using Bio-Rad Counting Slides (Bio-Rad#1450020) and the Bio-Rad TC20-Automated-Cell-Counter. 0.5 × 10^6^ cells were resuspended in 100 μL of AnnexinV Binding Buffer, transferred to round-bottom polystyrene tubes (Falcon#149596), and stained with 2 μL APC-AnnexinV and 1.5uL Propidium Iodide for 15 min. Data acquisition was performed using the four-laser LSR-Fortessa from BD, operated with the BD DIVA software. Data were analyzed using FlowJo V10 software.

### RNA Immunoprecipitation

RNA immunoprecipitation of plasmid transfected HeLa cells was performed 24 h after transfection, and 1.5 h after STS treatment. Cells were lysed as described above and 1 μl of RNaseOUT Ribonuclease Inhibitor (Invitrogen#10777-019) was added to each sample. Before immunoprecipitation, 2.5% of extract was kept to assess Input RNA. The GFP fusion proteins were immunoprecipitated as previously described [51] with Sera-Mag Protein A/G beads (Cytiva) and GFP antibody. After elution, RNA was extracted using TRIzol (Invitrogen#15596026) extraction method as recommended by the manufacturer. RNA was reverse transcribed using 5X iScript reagent (BioRad). mRNA levels of *XIAP* and *BCL-2* were assessed by RT-qPCR (ThermoFisher Scientific#4368577). Relative gene expression was normalized to GFP-transfected cells.

Primers used for RT-qPCR:

XIAP(F:5′-TGAATCTGATGCTGTGAGTT-3′, R:5′-CCTCAAGTGAATGAGTTAAA-3′),

BCL-2(F:5′-GGATGCCTTTGTGGAACTGT-3′, R:5′-AGCCTGCAGCTTTGTTTCAT-3′).

### Statistical analysis and data visualization

Statistical analysis was done using GraphPad V.10 Software. Briefly, to compare the difference in means between two groups, unpaired *T*-test was performed. For more than two groups, one-way ANOVA with Tukey’s multiple comparison tests was used. Error bars represent means and ±SEM of at least two independent experimental replicates with *P* ≤ 0.05, **P ≤ 0.01, ***P ≤ 0.001, and ****P ≤ 0.0001. ns not significant.

## Supplementary information

Supplementary Figure Legends

Figure S1

Figure S2

Figure S3

Figure S4

Figure S5

Full Length Western Blots - Figure 1

Full Length Western Blots - Figure 2

Full Length Western Blots - Figure 3

Full Length Western Blots - Figure 4

Full Length Western Blots - Figure S1

Full Length Western Blots - Figure S2

Full Length Western Blots - Figure S5

## Data Availability

All data generated during this study are included in this published article and its supplementary files.
