# Supplementary material for: An HuR mutant, HuR-V225I, identified in adult T-cell Leukemia/Lymphoma, alters the pro-apoptotic function of HuR
Source: Cell Death Discov. 2024 Dec 18;10:503. doi: 10.1038/s41420-024-02268-w (PMC11655865; doi:10.1038/s41420-024-02268-w)
Supplement: Supplementary file 1 — Supplementary Figure Legends [file 41420_2024_2268_MOESM1_ESM.docx]

**Supplemental Figure Legends**

**Fig. S1: Staurosporine and Doxorubicin induce apoptosis in HeLa cells**

**A** Phase contrast images of HeLa cells treated with 1 uM STS for 3h. Magnification 10X and 20X. **B** (Left) Representative immunofluorescent images of DAPI-stained HeLa cells treated with 1 uM STS for 3h. The bottom images are in grayscale. Magnification 63X. (Right) Analysis of immunofluorescent images quantifying the percentage of cells undergoing nuclear blebbing. **C** Western blot analysis of HeLa cells treated with 1 uM STS or equivalent volume of DMSO for 3h. Immunoblotting was performed using anti-PARP1, anti-cleaved PARP1, anti-cleaved caspase-3 and anti-HuR. Endogenous HuR cleavage product (CP1) is overexposed. Total protein content (bottom) was used as loading control. **D** Quantification of HuR, PARP1, and Caspase-3 cleavage products (CPs) of HeLa cells treated with 1uM STS, relative to total protein, normalized to untreated cells. **E** Western blot analysis of HeLa cells treated with 0-8 uM doxorubicin or equivalent volume of water for 18h. Immunoblotting was performed using anti-PARP1, anti-cleaved PARP1, anti-cleaved caspase-3 and anti-HuR. Endogenous HuR cleavage product (CP1) is overexposed. Total protein content (bottom) was used as loading control. **F** Quantification of HuR, PARP1, and Caspase-3 cleavage products (CPs) of HeLa cells treated with doxorubicin, relative to total protein, normalized to untreated cells. Error bars represent means and ± SEM of at least three independent experimental replicates with **P* ≤ 0.05, ***P* ≤ 0.01. ns, not significant by Unpaired t-test.

**Fig. S2: HuR-V225I is cleaved less than wild-type HuR in SH-SY5Y neuroblastoma cells in response to STS-induced apoptosis**

**A** Western blot analysis of SH-SY5Y cells treated with 1 uM STS for 1.5h. Immunoblotting was performed using anti-PARP1, anti-cleaved PARP1, anti-cleaved caspase-3 and anti-HuR. **B** Quantification of HuR, PARP1, and Caspase-3 cleavage products (CPs) of SH-SY5Y cells treated with 1uM STS, relative to total protein and normalized to untreated cells. **C** Western blot analysis of SH-SY5Y neuroblastoma cells transfected with GFP or GFP-HuR fusion proteins treated with 1 uM STS for 1.5h. Immunoblotting was performed using anti-GFP. **D** Quantification of the ratio of cleavage products of GFP-HuR fusion proteins in A. relative to their full-length counterparts, normalized to non-treated GFP-HuR. Error bars represent means and ± SEM of at least three independent experimental replicates with **P* ≤ 0.05, ***P* ≤ 0.01, ****P* ≤ 0.001. ns, not significant by Unpaired t-test (B) One-Way ANOVA (D).

**Fig. S3: HuR and HuR-V225I remain nuclear under non-treated conditions**

**A** Representative immunofluorescent images of non-treated HeLa cells transfected with GFP-HuR or GFP-HuR-V225I. Cells were stained with DAPI (nuclei), G3BP1 (cytoplasm) and GFP (HuR fusion proteins). Magnification 63X. **B** Quantification of nuclear and cytoplasmic fluorescence intensities of GFP-HuR vs. GFP-HuR-V225I in non-treated conditions. No changes in fluorescence intensities. Nuclear and cytoplasmic mean fluorescence intensities of GFP-HuR fusion proteins were quantified using Human C-N Translocation pipeline from Cell Profiler Software. Error bars represent means ± SEM of at least three independent experimental replicates with ns, not significant by Unpaired t-test.

**Fig. S4: HuR-D226A does not shuttle to the cytoplasm in response to STS-induced apoptosis in HeLa cells**

**A** Representative immunofluorescent images of HeLa cells transfected with GFP-HuR or GFP-HuR-D226A treated with 1 uM STS or equivalent volume of DMSO for 1.5h. Cells were stained with DAPI (nuclei), G3BP1 (cytoplasm) and GFP (HuR fusion proteins). Magnification 63X. **B** Quantification of nuclear and cytoplasmic fluorescence intensities of GFP-HuR vs. GFP-HuR-D226A in DMSO-treated conditions. No changes in fluorescence intensities. **C** Quantification of nuclear and cytoplasmic fluorescence intensities of GFP-HuR vs. GFP-HuR-D226A in response to 1 uM STS for 1.5h. Nuclear and cytoplasmic mean fluorescence intensities of GFP-HuR fusion proteins were quantified using Human C-N Translocation pipeline from Cell Profiler Software. Error bars represent means ± SEM of at least three independent experimental replicates with *****P* ≤ 0.0001. ns, not significant by Unpaired t-tests.

**Fig. S5: PHA and STS induce apoptosis in T-lymphocytes**

**A** Western blot analysis of Jurkat T-lymphocytes treated with 2-10 ug/mL of phytohemagglutinin (PHA) for 24h. Immunoblotting was performed using anti-PARP1 anti-cleaved PARP1,anti-cleaved caspase-3 and anti-HuR. Total protein content (bottom) was used as loading control. **B** Quantification of HuR, PARP1, and caspase-3 cleavage products (CPs) of Jurkat cells treated with PHA, relative to total protein and normalized to untreated cells. **C** Western blot analysis of Molt-4 T-lymphocytes treated with 1 uM STS or equivalent volume of DMSO for 3h. Immunoblotting was performed using anti-PARP1, anti-cleaved PARP1, anti-cleaved caspase-3 and anti-HuR. Endogenous HuR cleavage product (CP1) is overexposed. Total protein content (bottom) was used as loading control. **D** Quantification of HuR, PARP1, and caspase-3 cleavage products (CPs) of Molt-4 cells treated with 1uM STS, relative to total protein and normalized to untreated cells. Error bars represent means ± SEM of at least three independent experimental replicates with **P* ≤ 0.05, ***P* ≤ 0.01, ****P* ≤ 0.001, and ns, not significant by Unpaired t-tests.
