# Supplementary figures and images for: An HuR mutant, HuR-V225I, identified in adult T-cell Leukemia/Lymphoma, alters the pro-apoptotic function of HuR
Source: Cell Death Discov. 2024 Dec 18;10:503. doi: 10.1038/s41420-024-02268-w (PMC11655865; doi:10.1038/s41420-024-02268-w)

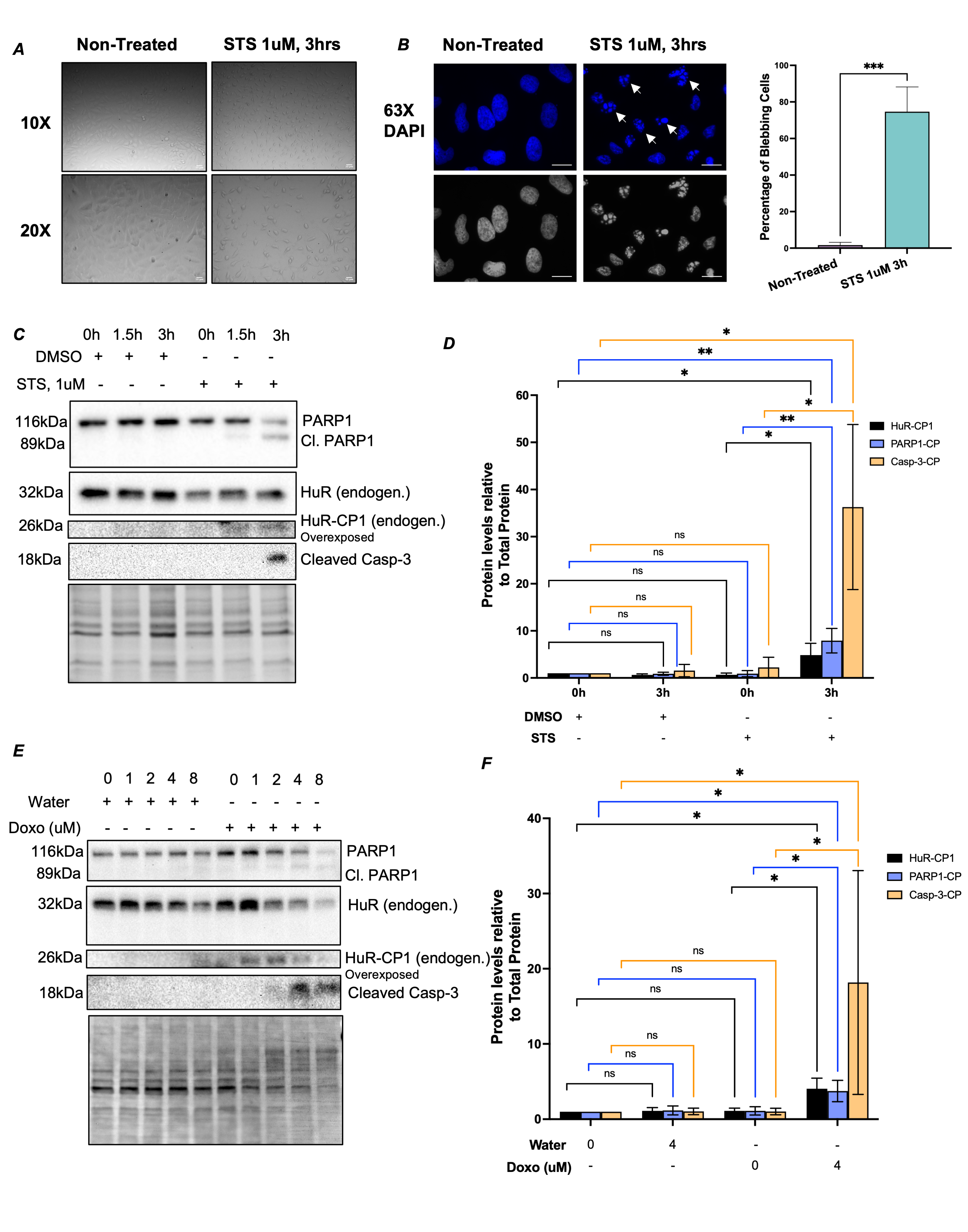

Supplement: Supplementary file 2 — Figure S1 [file 41420_2024_2268_MOESM2_ESM.png]

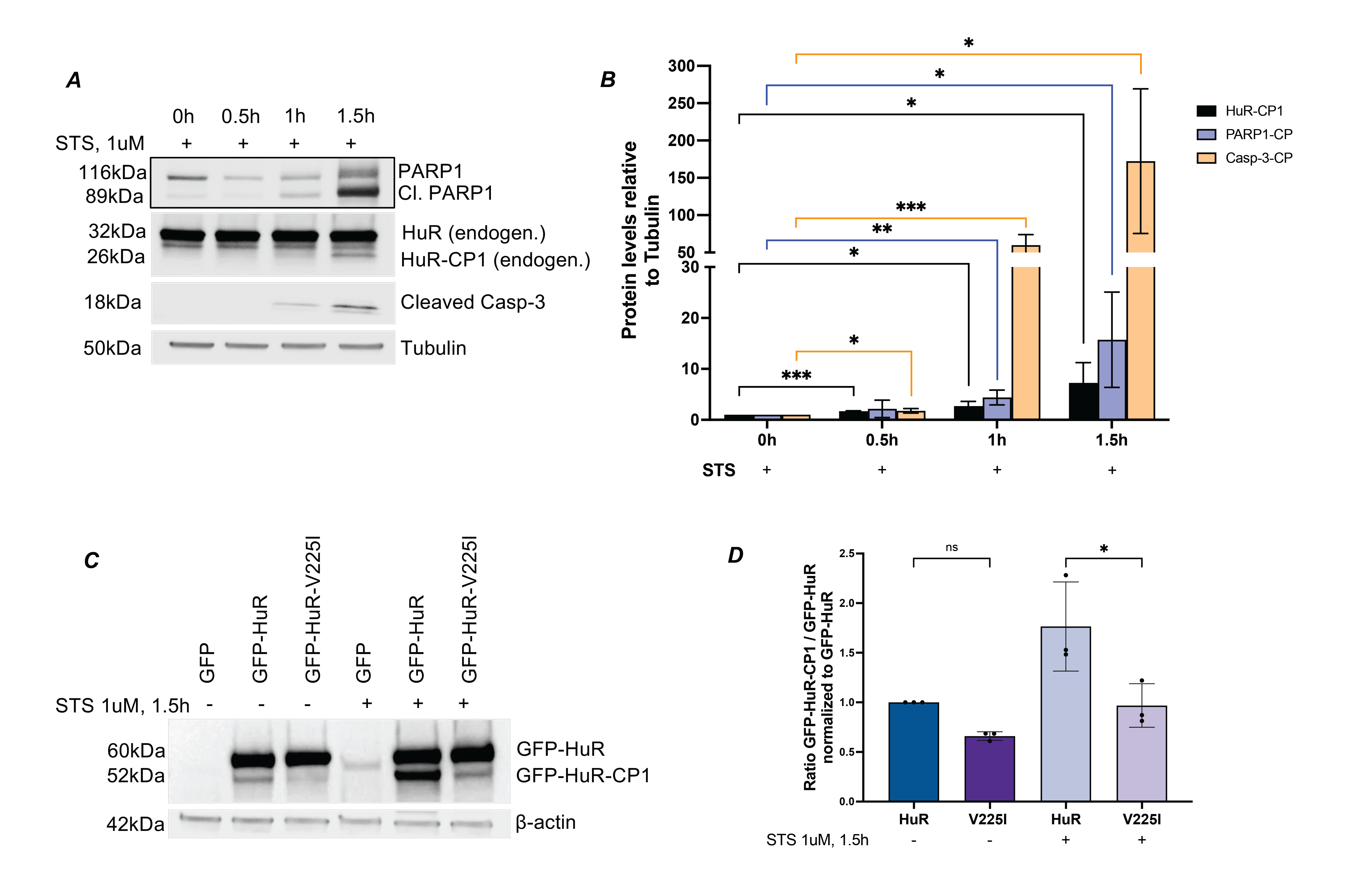

Supplement: Supplementary file 3 — Figure S2 [file 41420_2024_2268_MOESM3_ESM.png]

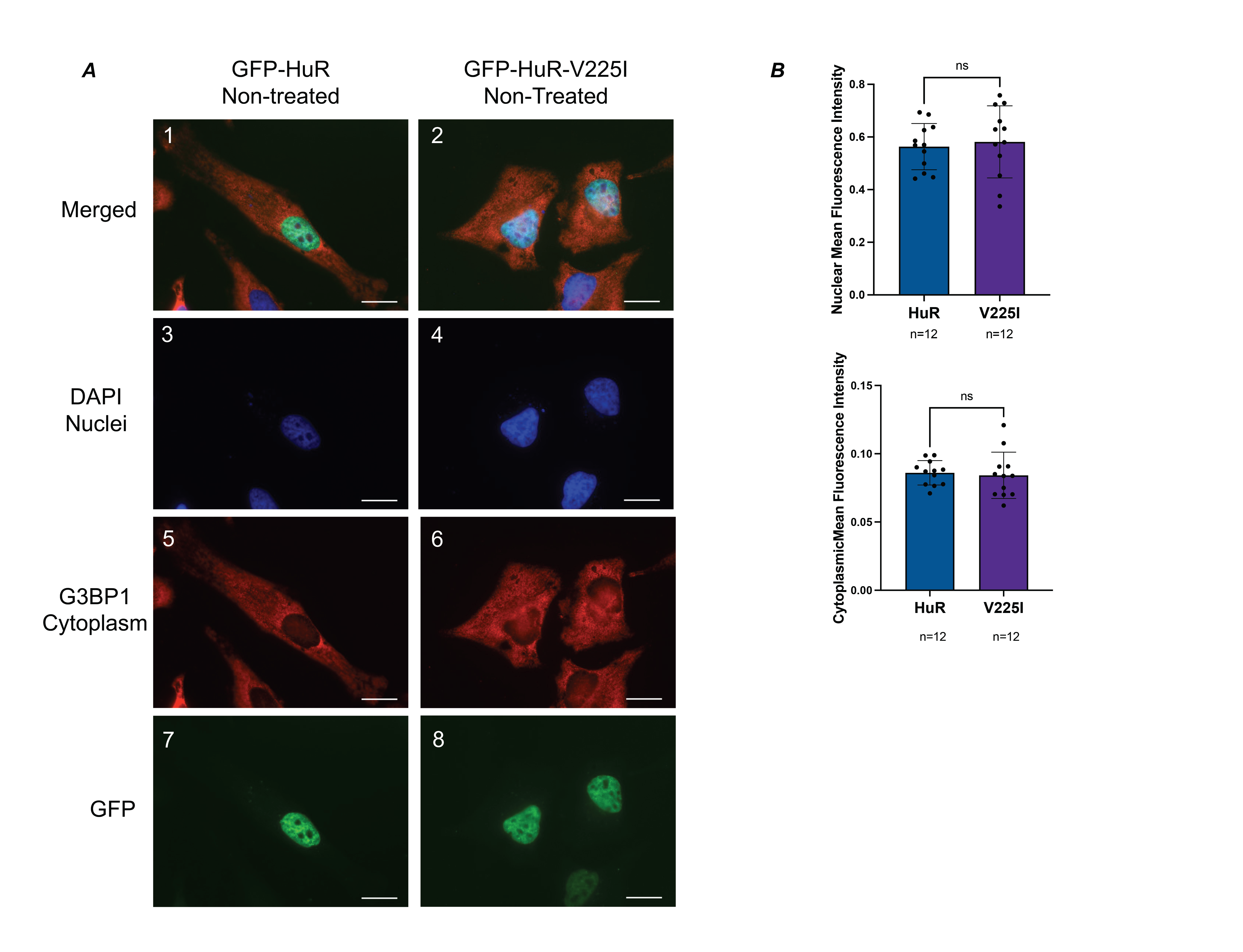

Supplement: Supplementary file 4 — Figure S3 [file 41420_2024_2268_MOESM4_ESM.png]

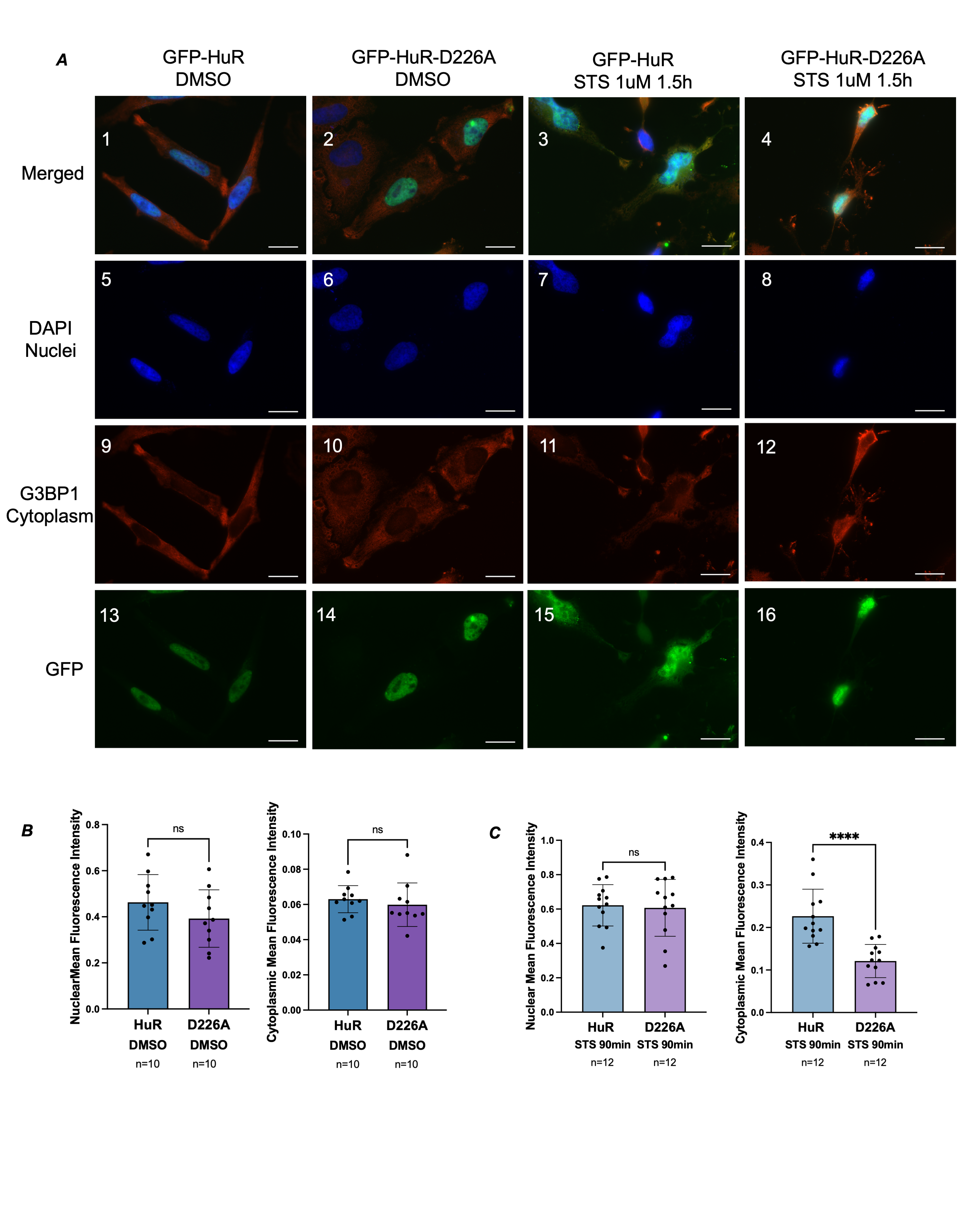

Supplement: Supplementary file 5 — Figure S4 [file 41420_2024_2268_MOESM5_ESM.png]

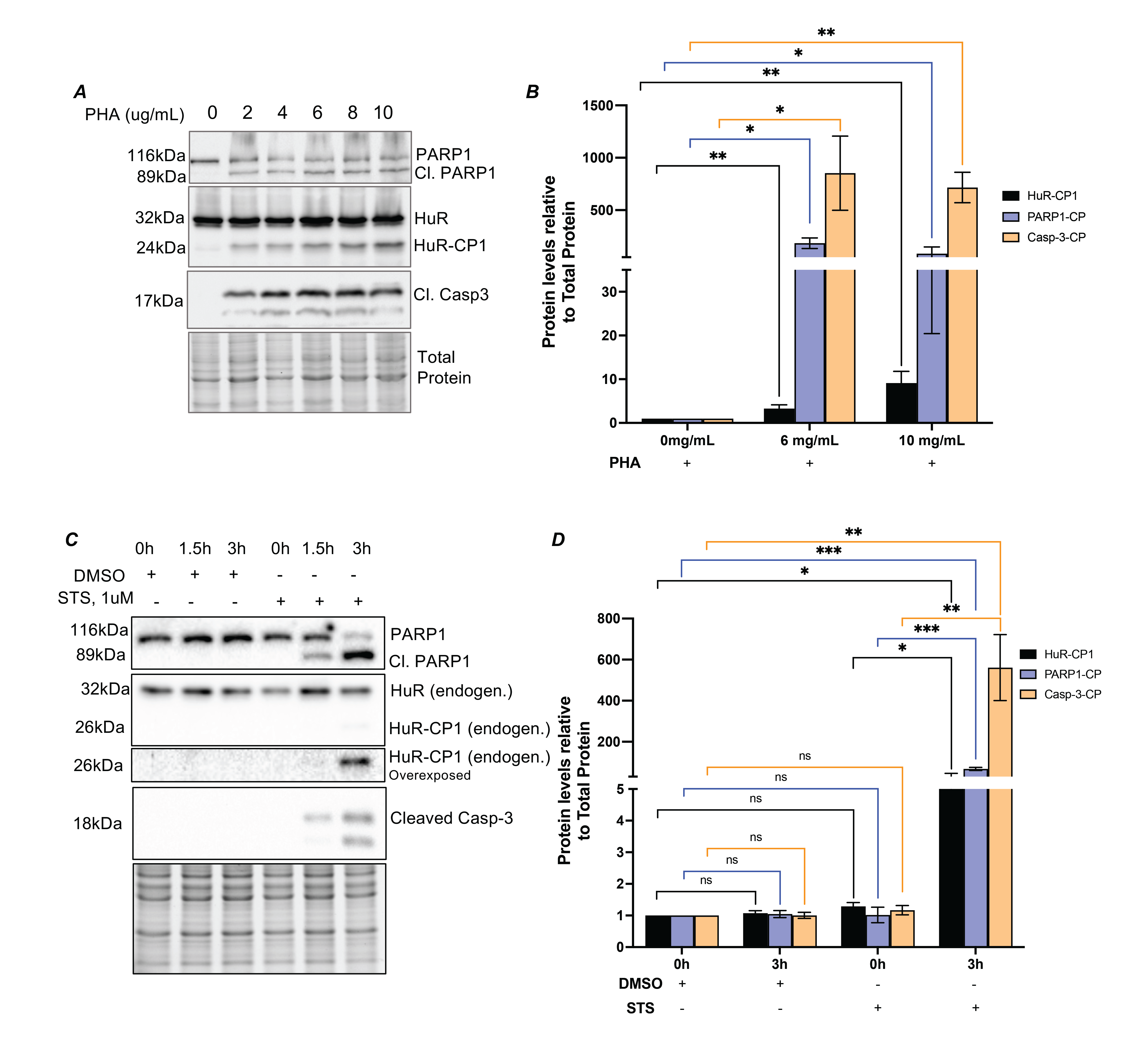

Supplement: Supplementary file 6 — Figure S5 [file 41420_2024_2268_MOESM6_ESM.png]

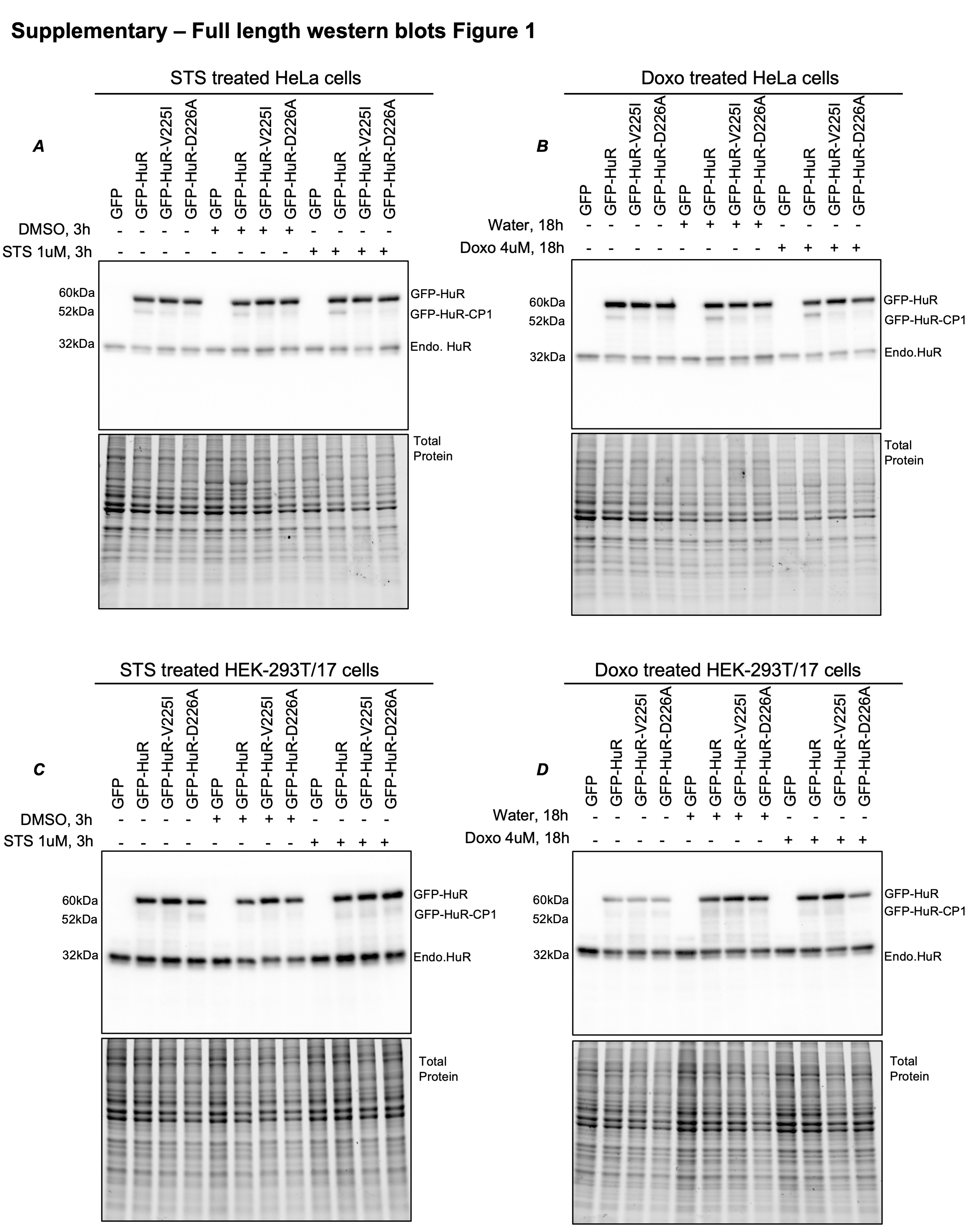

Supplement: Supplementary file 7 — Full Length Western Blots - Figure 1 [file 41420_2024_2268_MOESM7_ESM.png]

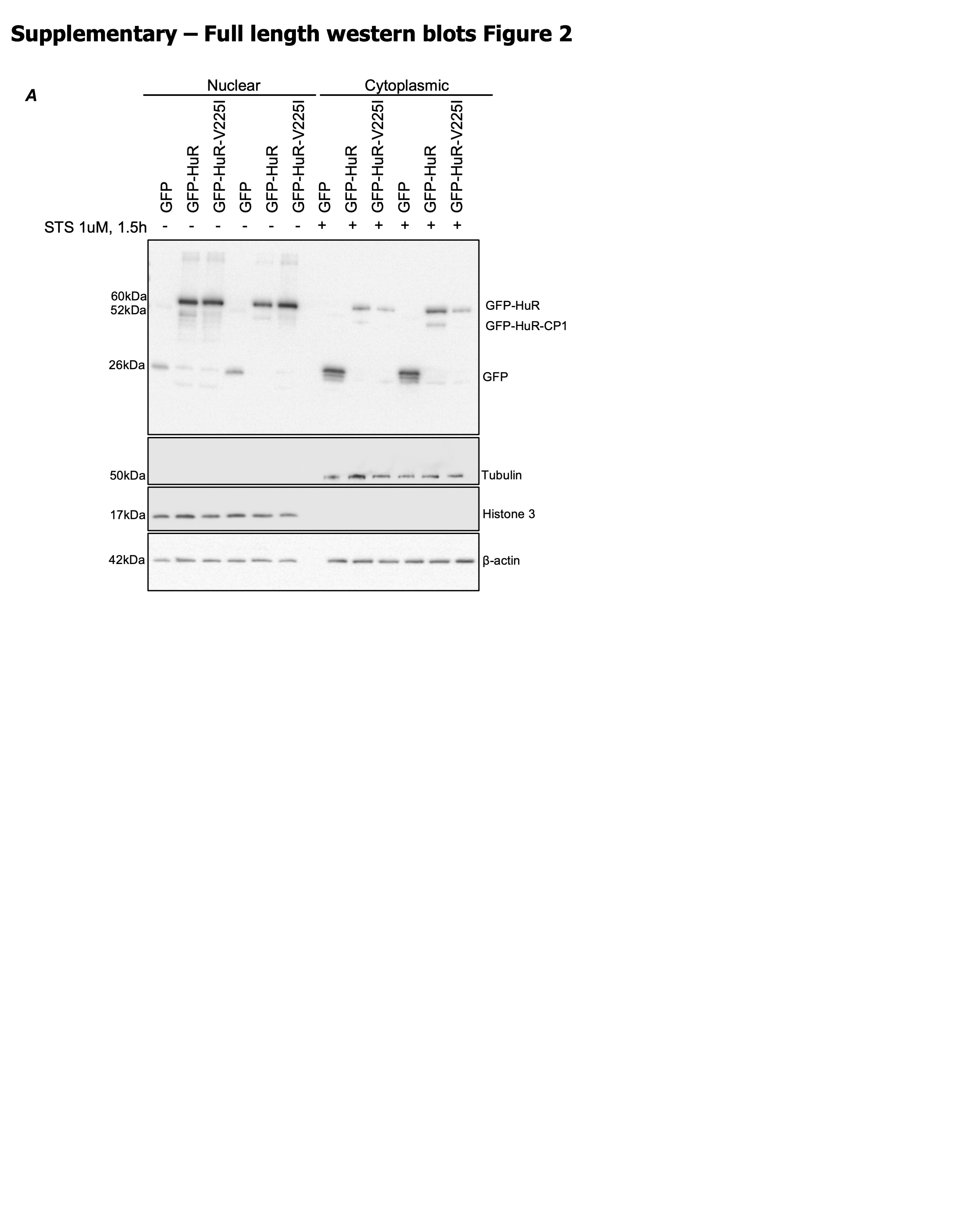

Supplement: Supplementary file 8 — Full Length Western Blots - Figure 2 [file 41420_2024_2268_MOESM8_ESM.png]

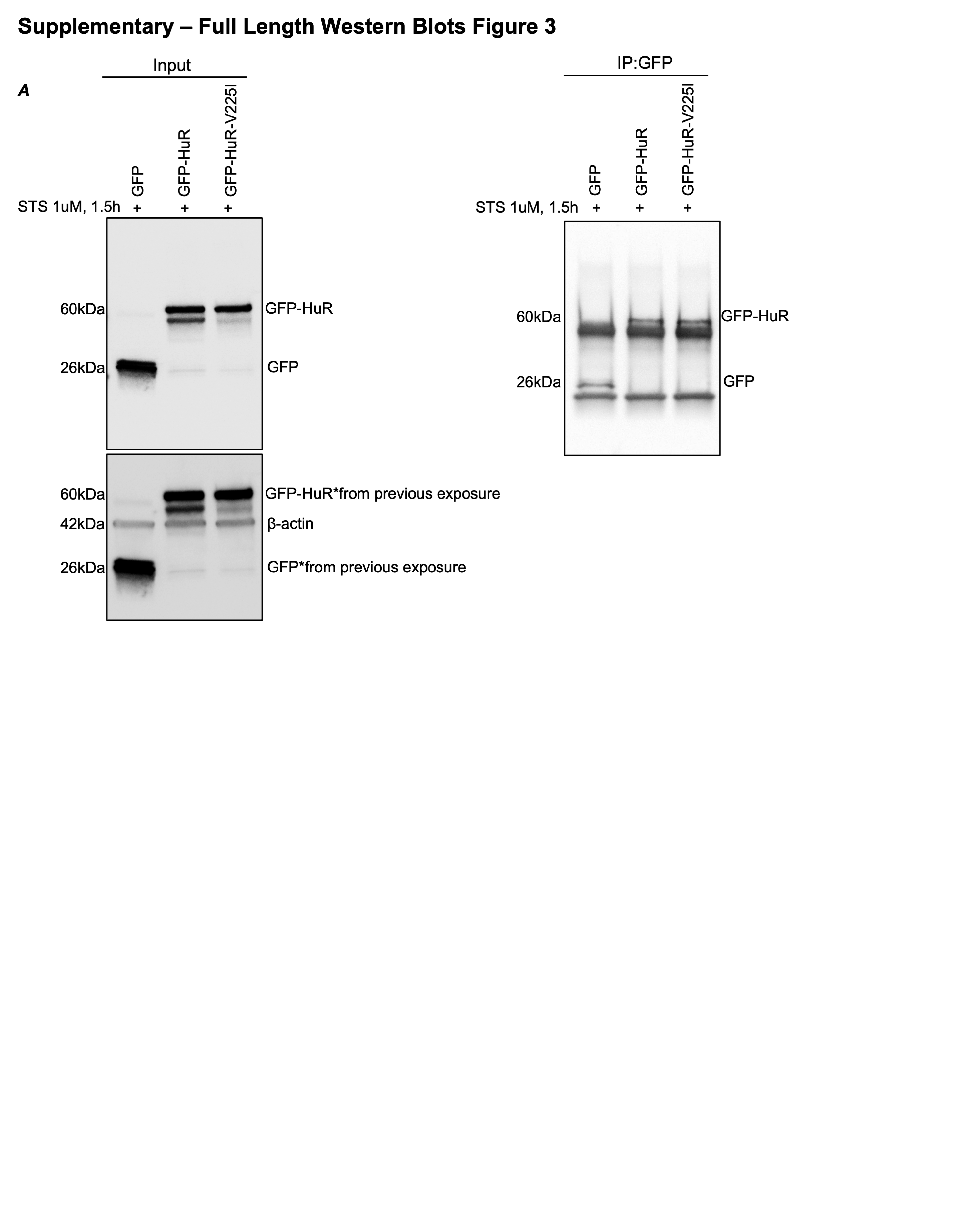

Supplement: Supplementary file 9 — Full Length Western Blots - Figure 3 [file 41420_2024_2268_MOESM9_ESM.png]

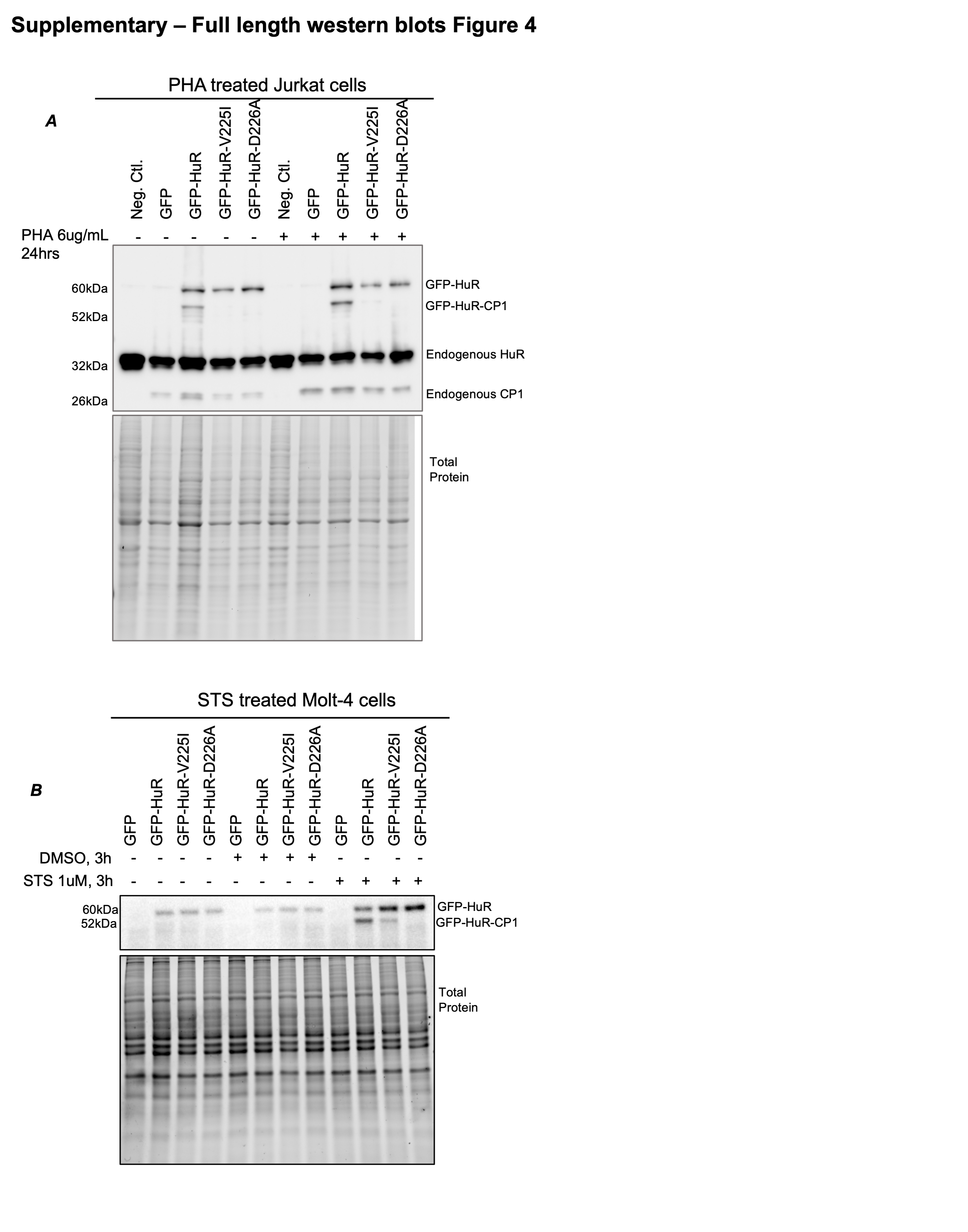

Supplement: Supplementary file 10 — Full Length Western Blots - Figure 4 [file 41420_2024_2268_MOESM10_ESM.png]

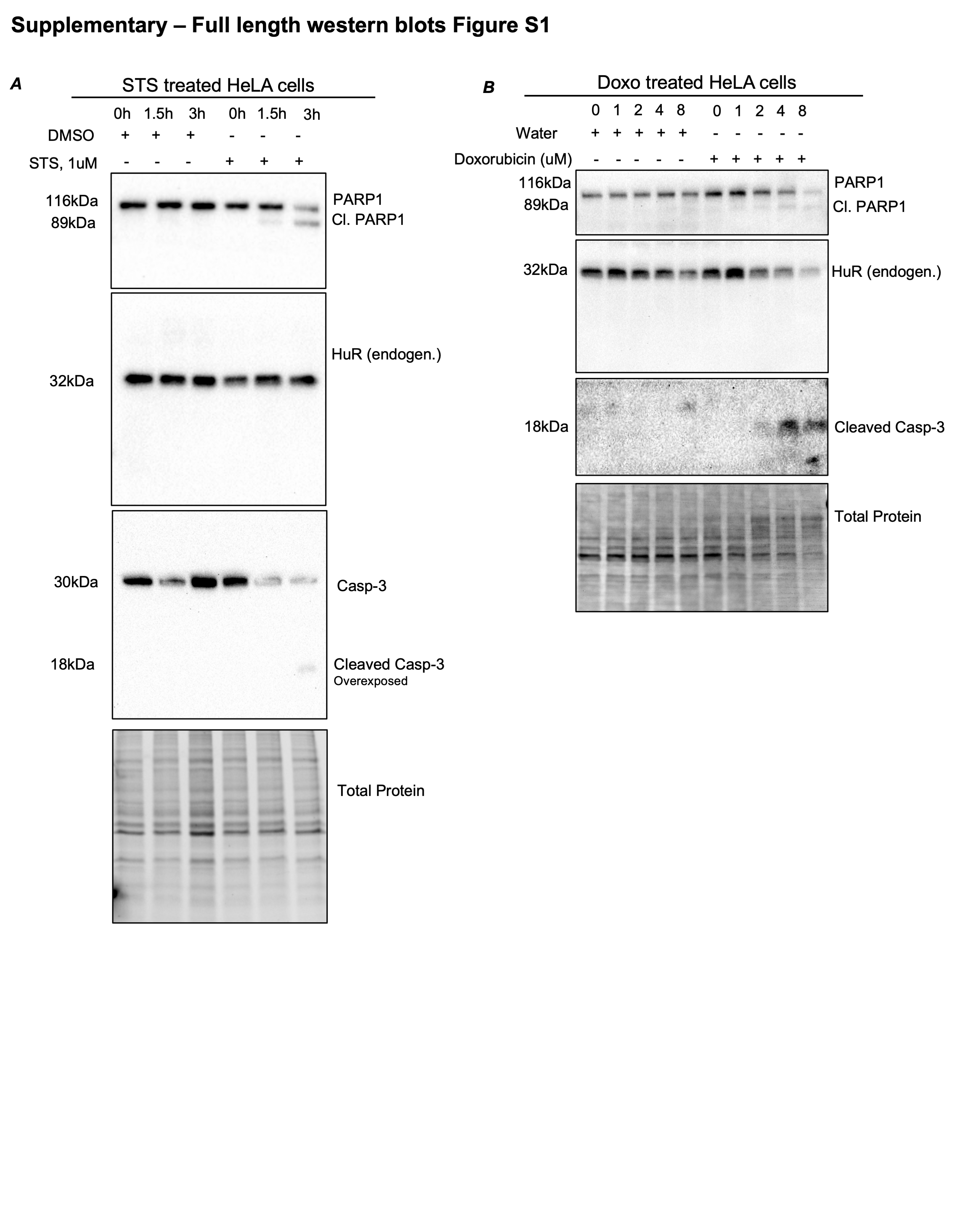

Supplement: Supplementary file 11 — Full Length Western Blots - Figure S1 [file 41420_2024_2268_MOESM11_ESM.png]

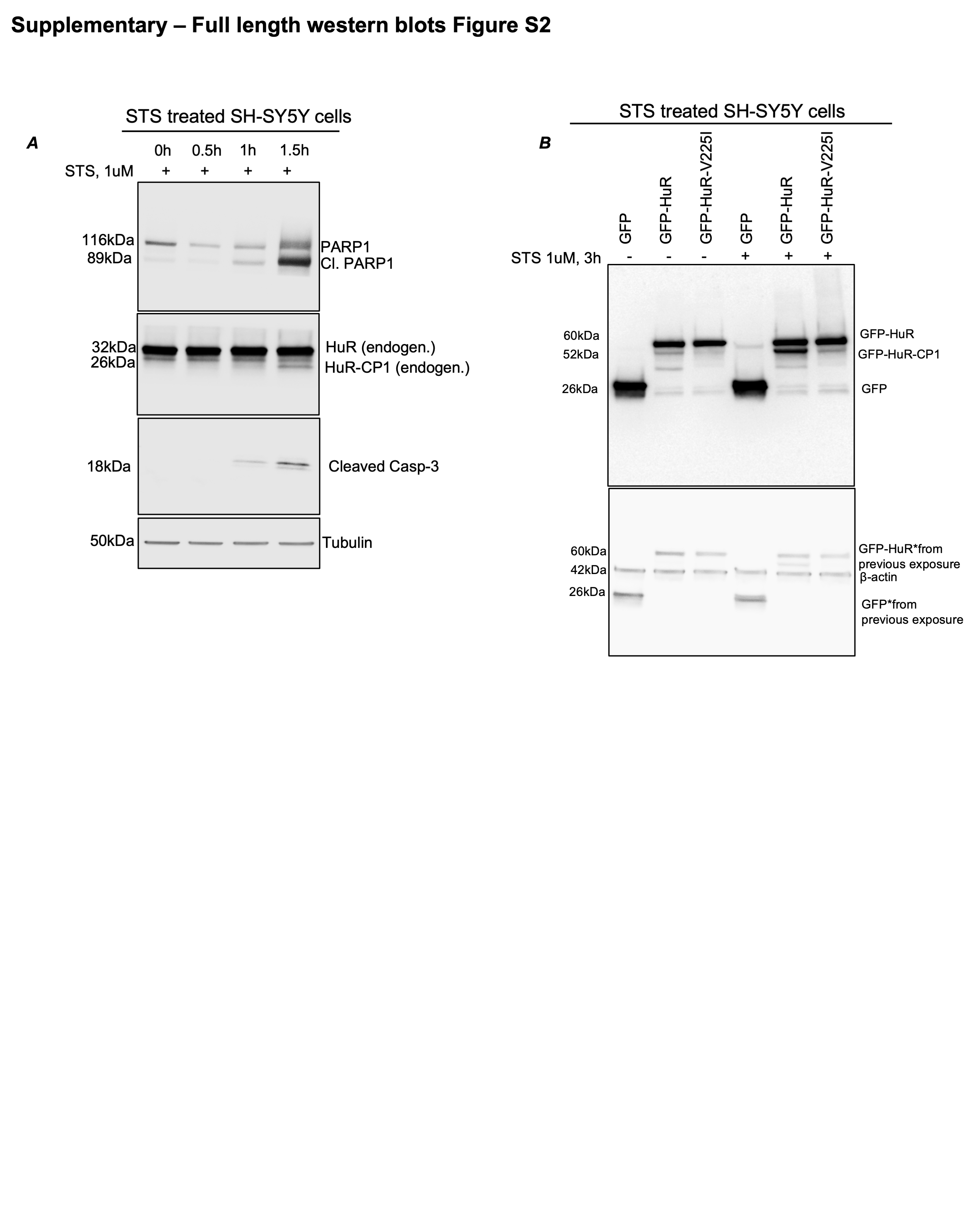

Supplement: Supplementary file 12 — Full Length Western Blots - Figure S2 [file 41420_2024_2268_MOESM12_ESM.png]

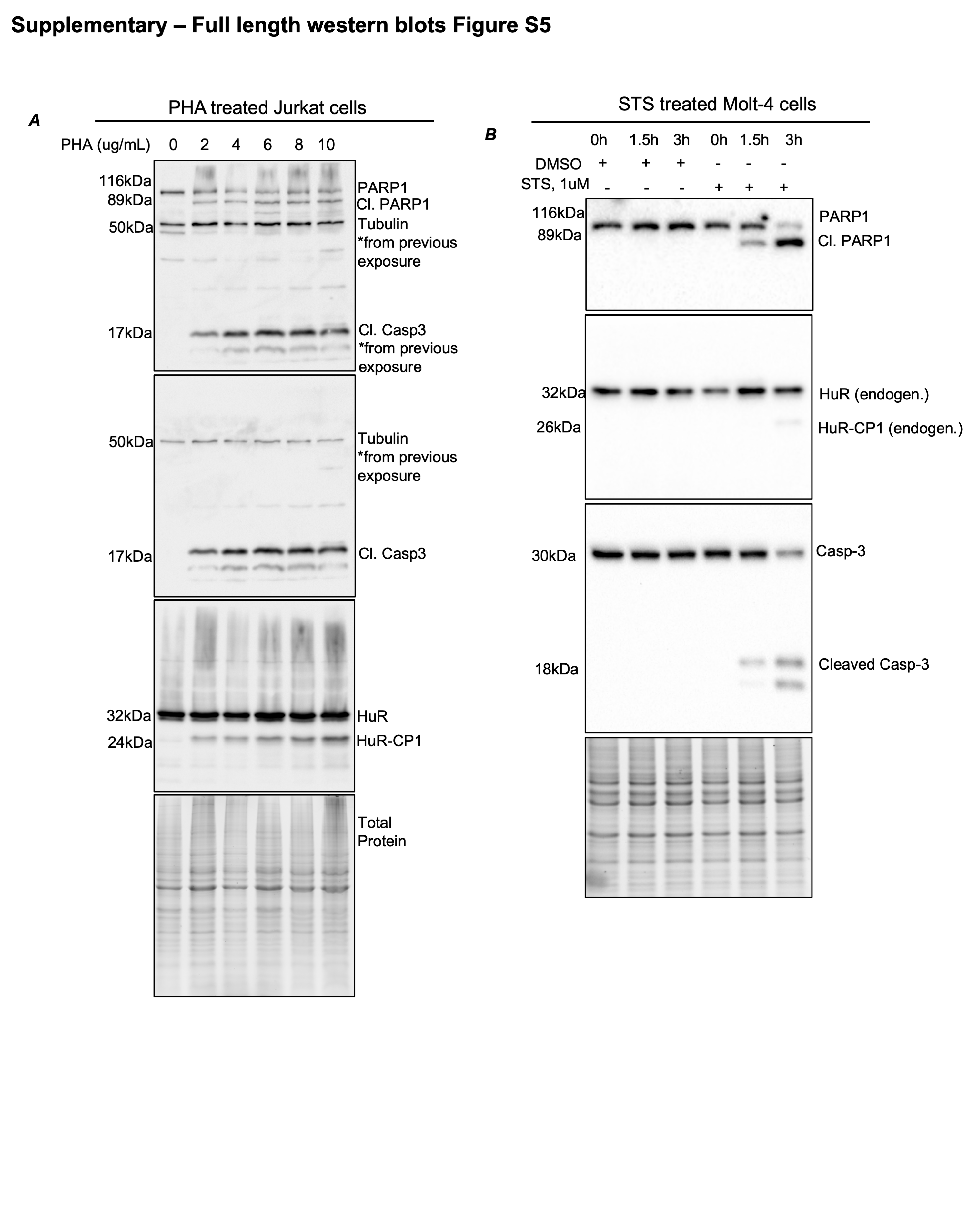

Supplement: Supplementary file 13 — Full Length Western Blots - Figure S5 [file 41420_2024_2268_MOESM13_ESM.png]
